# Isolation of vaccinia JX594 from pustules following therapy for hepatocellular carcinoma

**DOI:** 10.1186/s12885-015-1753-4

**Published:** 2015-10-15

**Authors:** Che-Hsuan Kung, Shu-Chen Kuo, Te-Li Chen, Wen-Sung Weng

**Affiliations:** 1 Division of Infectious Diseases, Department of medicine, Taipei Veterans General Hospital, Taipei, Taiwan; 2 Department of Internal Medicine, Taipei City Hospital, Zhongxing Branch, Taipei, Taiwan; 3 Department of Internal Medicine, Cheng Hsin General Hospital, Taipei, Taiwan; 4 Institute of Clinical Medicine, School of Medicine, National Yang-Ming University, Taipei, Taiwan; 5 National Institute of Infectious Diseases and Vaccinology, National Health Research Institutes, Miaoli County, Taiwan; 6 Division of Microbiology, Department of Pathology and Laboratory Medicine, Taipei Veterans General Hospital, Taipei, Taiwan

**Keywords:** JX594, Vaccinia poxvirus, Pustule, Hepatocellular carcinoma

## Abstract

**Background:**

JX594 is an oncolytic poxvirus derived from Wyeth strain vaccinia virus. We reported the presentation of cutaneous and mucosal pustules containing laboratory-confirmed JX594 in a patient following injection of JX594.

**Case presentation:**

A 36-year-old man was diagnosed hepatitis B virus-associated hepatocellular carcinoma on September 19, 2011. Despite treatment with entecavir, radiofrequency ablation and transarterial chemoembolization for recurrent local tumors, the tumors recurred in both lobes and lung metastases were detected by computed tomography on September 12, 2012. The patient was treated with JX594 (Pexa-vec®) via intravenous injection on December 19, 2012. No apparent adverse effects were observed following intravenous injection other than a single fever episode. However, pustular lesions were detected on both sides of the tongue dorsum and on the proximal interphalangeal joint of the right middle finger on December 25, 1012. Biopsy samples analyzed by PCR identified the presence of the JX-594-specific hGM-CSF transgene and the disrupted viral thymidine kinase gene. Following aspiration of the lesion a scab formed that resolved within 14 days without necessitating additional treatment.

**Conclusion:**

Our case completely recovered and did not develop systemic or recurrent disease, the presentation of a few pustules may not necessarily require that treatment with JX594 be interrupted for patients with advanced hepatocellular carcinoma.

## Background

JX594 is an oncolytic poxvirus derived from the Wyeth strain vaccinia virus [1]. Various modifications to JX594, including the interruption of thymidine kinase, dependency on epidermal growth factor receptor-ras pathway, and resistance to interferon allow it to selectively replicate within tumor cells [2]. Lysis of vaccinia virus-infected tumor cells results as a consequence of viral replication combined with tumor specific immunity that is enhanced by JX594-derived granulocyte macrophage-colony stimulating factor (GM-CSF). Injection of JX594 into melanoma patients or patients presenting with advanced hepatic cancer either at the tumor site or systemically can result in tumor regression [3, 4]. Adverse effects reported following JX594 administration were relatively mild (i.e., flu-like symptoms) [4] and JX594 dissemination to distant sites other than the target tumor have been rarely reported. The present case report describes a patient presenting with advanced hepatocellular carcinoma that was treated with an intravenous injection of JX594 prior to developing distant skin and mucosal lesions containing laboratory-confirmed JX594 virus.

## Case presentation

The diagnosis of hepatitis B virus (HBV)-associated hepatocellular carcinoma was confirmed by pathology on a 36-year old male patient on September 19, 2011. Despite treatment with entecavir, radiofrequency ablation (September 29, 2011 and May 10, 2012), and transarterial chemoembolization (July 30, 2012) for recurrent local tumors, the tumors recurred in both lobes and lung metastases were detected by computed tomography on September 12, 2012.

**Fig. 1:**
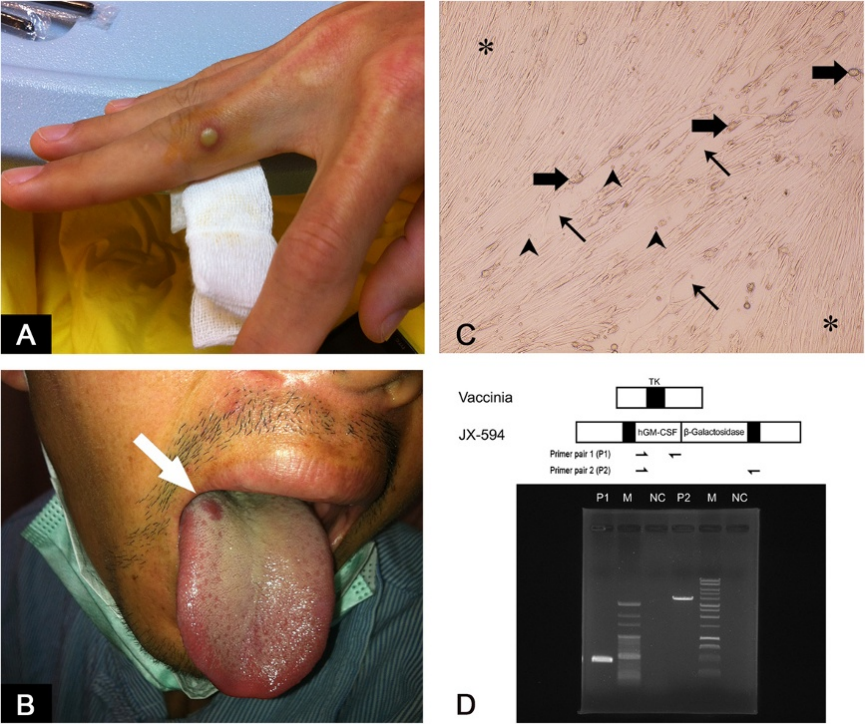
**a** The patient developed a pustular lesion on the proximal interphalangeal joint of the right middle fingerfollowing intravenous injection with JX594. **b** Other pustular lesions were noted on the both sides of the tongue dorsum. **c** The cytopathic effect was observed by co-culturing the virus with MRC-5 cells. Asterisk: normal MRC-5 cells structure; Thin arrow: discohesive structure; Bold arrow: rounded cell; Arrowhead: necrotic debris and vesicles. **d** PCR designed to amplify the JX-594-specific hGM-CSF transgene (Primer pair 1) and the disrupted viral thymidine kinase gene (Primer pair 2) was positive. M, marker; NC, negative control

Therefore, the patient was treated with an initial dose (10^9^ plaque forming unit [pfu]) of JX594 (Pexa-vec®) via intravenous injection on December 19, 2012 (ClinicalTrials.gov Identifier: NCT01387555). No apparent adverse effects were observed following intravenous injection other than a single fever episode. However, on December 25, 2012 pustular lesions were detected on the proximal interphalangeal joint of the right middle finger (Fig. 1a) and on both sides of the tongue dorsum (Fig. 1b). Physical examination did not reveal any additional skin or mucosal lesions. The patient’s contact history was unremarkable. The patient’s white blood cell count on the day the pustular lesions were identified were 7100 per cubic millimeter (61.1 % neutrophils, 21.7 % lymphocytes) and a hemoglobin concentration of 14.0 g/dl. Serology was negative for measles virus, varicella zoster, EB-VCA, rubella, and CMV. Pus aspirated from the pustules on December 28 was JX594 positive. The discharge was diluted and filtered using 0.45-μm-pre-size filter, and cocultured with MRC-5 (lung fibroblast cell line). The cells were then maintained in the minimal essential medium (Gibco, life technologies, USA), supplemented with 2 % fetal bovine serum and 1 % penicillin-streptomycin-gentamycin at 37 °C with 5 % CO_2_. The cytopathic effect, such as discohesive arrangement, cell rounding and shrinkage, and necrosis with vesicles and cell debris, was apparent (Fig. 1c). Biopsy samples analyzed by PCR identified the presence of the JX-594-specific hGM-CSF transgene (Primer pair 1) and the disrupted viral thymidine kinase (TK) gene (Primer pair 2, accession no. AF163844). The sequences amplified were 99 % similar to the hGM-CSF (accession no. M10663) and beta-galactosidase (accession no. U54829) sequences available on the NCBI database. Following aspiration of the lesion a scab formed that resolved within 14 days without necessitating additional treatment. The patient received intra-tumor injection on December 26, 2012 but there was no further skin or mucosal lesions detected on follow-up.

## Conclusions

Vaccination with vaccinia virus was used successfully as part of the smallpox eradication program with low adverse effects rates. Major complications such as generalized vaccinia were approximately 86.7–122.2 per million vaccinations [5]. Theoretically, this suggested that vaccinia virus engineered to replicate exclusively within tumor cells would also be associated with low complication rates. During a phase 1 trial, 1 case that received a high intratumor injection dose of JX594 (10^9^ PFU) developed multiple pustules 4 days latter [4], but this manifestation was not observed in other patients (during other clinical trials) injected with lower doses [3, 6, 7]. However, follow-up experiments designed to identify vaccina virus in the pustules were not performed in the suspected case [4]. In contrast, live virus was identified in the aspirates of the present case study and PCR analysis identified the presence of a disrupted thymidine kinase gene (associated with JX594) suggesting that JX594 replicated within the epithelial cells of the patient’s oral cavity and skin. These data suggested that the lesions discovered on December 25, 2012 were caused by the systemic spread of JX594 following intravenous injection on December 19, 2012.

Following small pox vaccination, papules typically present 4–5 days later and become pustular within 7–10 days. The pustules identified in our case (and on the previously described case [4]) appeared following the first dose of JX594 but did not recur. It is not known whether the neutralizing antibody which developed in almost all cases receiving JX594 may have a role in preventing subsequent pustule development considering the replication and good treatment efficacy of JX594 in the presence of high titer of neutralizing antibody in previous reports [4, 7]. Since our case and the previous suspected case [4] completely recovered and did not develop systemic or recurrent disease, the presentation of a few pustules may not necessarily require that treatment with JX594 be interrupted for patients with advanced hepatocellular carcinoma.

## Consent

Written informed consent was obtained from the patient for publication of this case report and any accompanying images. A copy of the written consent is available for review by the Editor of this journal.

## Abbreviations

GM-CSF: Granulocyte macrophage-colony stimulating factor
HBV: Hepatitis B virus
NCBI: National Center for Biotechnology Information
PFU: Plaque-forming unit
PCR: Polymerase chain reaction
